# Hydriodate of Potash

**Published:** 1844-07

**Authors:** 


*London Lancet.*

---

*Hydriodate of Potash.*—A writer in the London Lancet condemns the practice of giving this medicine in half ounce doses, which, he thinks, would prove fatal if the article were pure. He says:

Gr. x. *ter die*, is the largest dose that I have ever given, beginning with gr. iij. *ter die*, with the greatest success (internally) in cases of diseased joints. Externally I have used it with curative effects in form of ointment, thus:—℞ Hydriod. pot. ʒj.; aq. distill. ʒj. Solve and mix with ℥j. of lard; a piece of the size of a large nut, to be used two or three times a day. I have at this moment a case of bronchocele which is progressing favorably under the use of the remedy; the patient has come from Gravesend to be under my care. She was all but suffocated, and left to die, after consultations repeated during eleven or twelve years. I believe I shall cure her.

